# Abstracts from Foreign Journals

**Published:** 1901-05

**Authors:** M. P. Ravenel, S. J. J. Harger

**Affiliations:** Philadelphia, Pa.; Philadelphia, Pa.


### ABSTRACTS FROM FOREIGN JOURNALS.

### FRENCH.

Under the Direction of M. P. Ravenel, M.D., and S. J. J. Harger, V.M.D., of Philadelphia, Pa.

Persistence of the Genesic Instinct after Incomplete Castration. Instances of this kind have often been noticed, and old castrators make a distinction between "high" and "low" castration upon the future disposition of the animal. M. Bournay reports two such cases. In one of them the horse showed the characters of the stallion in the head, neck, sexual desire on the approach of mares, erection of the penis, and, finally, he became very vicious. Two cicatrices of the scrotum indicated previous castration. The end of the cord was as large as a walnut. The inguinal portion was larger than in the gelding. The animal was recastrated and became docile. Examination of the enlarged cord showed that it contained the epididymis, but no trace of the testicular tissue itself. In the other case the same treatment was successful. Bournay calls attention to the benefit of surgical intervention in cases of vicious character coincident with excessive volume of the testicular cord.—*Revue Vétérinaire.*

Treatment of Demodectic Mange in the Dog. Roth's treatment of demodectic mange in the dog was applied to fifteen dogs at the Leipzig University. This consists in shaving off with a razor the epidermis of the affected skin until small drops of blood freely ooze through the skin. The parts are then covered with a dressing saturated with corrosive sublimate, and after several days are dressed with iodoform and boric acid. The diseased patches varied in size from that of one to five franc pieces. The diagnosis was made with the microscope. Of the fifteen cases thirteen were cured, eleven after the first and two after the second operation. The treatment is efficacious when the disease is limited. The parasites, which are not removed with the instrument, are destroyed by the antiseptics or by simple desiccation.—*Revue Vétérinaire.*

Primary Ostitis of the Os Pedis. In discoursing upon ostitis and exostoses, Jacoulet describes a pathologic process of the os pedis frequently met but often misinterpreted. He describes ostitis of the third phalanx, a lesion which commences in an obscure manner and is indicated at first only by a slight modification in the position of the leg, the manner of its movement, and a slight abnormal heat of foot. Other symptoms soon appear; the posterior and the coronet soon become straight, and the foot contracted. In other cases the foot assumes a form as seen in chronic laminitis. This alteration is designated subacute founder (sub-fourbure) in order to distinguish it from chronic founder which follows the acute form of the disease. These phenomena are attributed to a progressive ostitis of the anterior face of the third phalanx, determining alterations of the podophyllous and prophyllous tissue. The author reports cases upon this point. He concludes: Primary ostitis of the third phalanx is often a primary cause of numerous lamenesses, obscure at the beginning, which may not have any external characteristic lesions, or, if the ostitis is moderate, may result in marked contraction of the foot, and, if intense, in chronic founder.—*Annales de Méd. Vét.*

Can the Ass Contract Tuberculosis? Galtier, from his experiments, claims the following results: That the ass, highly resisting to tuberculosis, can become infected spontaneously as well as experimentally, especially if the inoculation be intravenous (jugular) and obtained from the rabbit or the guinea-pig destroyed by bovine tuberculosis; that the result of such injection, according to the dose, may be fatal, remain latent, or terminate in recovery; that the lesions resulting from this mode of infection are principally found in the lungs, in which they take the form of nodules or homogeneous granulations of the size of a millet-seed to that of a haricot, and that these become calcareous when the disease terminates in recovery.—*Annales de Méd. Vét.*

Chloral as an Anæsthetic in the Horse. According to Fröhner, chloroform is far from being an inoffensive anæsthetic. Upon 800 horses anæsthetized he has observed one case of poisoning from a dose of 55 grammes; one case of asphyxia with 48 grammes; two cases of pulmonary gangrene; several cases of vomiting; arrest of respiration, and acute paralysis of the vocal cords.

Morphine is less dangerous to the horse, of easy employment hypodermatically, and may render some practical value, but it produces only a mild narcosis, which is sometimes replaced by a state of excitation which lasts for several hours after the operation; it is not rare to see regurgitation and vomiting followed by pneumonia from error in swallowing.

Fröhner substituted chloral for chloroform and morphine in the form of a clyster in seventy-five operations in the horse. In order to prevent irritation of the rectal mucous membrane the following formula is used: ℞.—Chloral hyd., 150 grammes; gum arab., 75 grammes; water, 3000 grammes.—M. To produce narcosis 100 grammes of chloral to 10 grammes per 100 kilos of body weight are administered. The injection is given about two hours before the operation, and preferably at the place where it is to be performed. The muscular and psychic depression continue from one to two hours; occasionally regurgitation and vomiting were seen. The vascular paralysis favoring hemorrhage was not noticed. In one case prolapsus of the rectum followed, but this was attributed to the solution being five days old and exposure to the sun; also, in one case œdema of the glottis occurred, requiring tracheotomy. The employment of chloral as a clyster and morphine hypodermatically is not recommended.

To obtain complete anæsthesia the chloral clyster may be supplemented by inhalations of chloroform.—*Monats. f. prak. Thierheilk.*

